# Quantitative analysis of the association between *CRP* rs2808630 and rs1417938 polymorphisms and cancer risk

**DOI:** 10.3892/ol.2014.2796

**Published:** 2014-12-12

**Authors:** JIAN-GONG WANG, YANG ZHANG, TIAN-LIN XIAO

**Affiliations:** 1 Department of Cancer Biotherapy, Cancer Institute, Tangshan People’s Hospital, Tangshan, Hebei 063001, P.R. China; 2 Department of Oncology, Xiangyang Central Hospital, Medical College, Hubei University of Arts and Science, Xiangyang, Hubei 441000, P.R. China

**Keywords:** C-reactive protein, polymorphism, cancer, meta-analysis

## Abstract

Accumulating evidence indicates that polymorphisms in the *CRP* gene are important in the development of cancer. The current meta-analysis was performed to investigate the association between *CRP* polymorphisms 3407 A>G (rs2808630) and 29 A>T (rs1417938), and the risk of developing cancer. A search of the relevant literature was conducted using the PubMed database to identify eligible studies published up until March 25, 2014. Five case-control studies involving 888 cases and 3,167 controls for the 3407 A>G polymorphism, and six case-control studies involving 3,110 cases and 5,951 controls for the 29 A>T polymorphism were included in the current meta-analysis. The pooled odds ratios with 95% confidence intervals were calculated using the fixed- or random-effects model. Meta-analysis identified no association between the *CRP* 3407 A>G and 29 A>T polymorphisms, and overall cancer risk. Additional stratified analysis by cancer type did not reveal any significant associations in the genetic models investigated. The findings of the present study indicated that *CRP* 3407 A>G and 29 A>T polymorphisms are not associated with cancer risk.

## Introduction

Chronic inflammation is important in the development of cancer at a number of sites in the body (1). C-reactive protein (CRP) belongs to the family of acute-phase proteins and, as a general marker for inflammation, is synthesized in hepatocytes. A major role of CRP is to bind phosphocholine, thereby permitting the recognition of foreign pathogens and the phospholipid constituents of damaged cells; thus, CRP is vital in host defense and the clearance of necrotic and apoptotic cells (2).

The *CRP* gene is located on chromosome 1q21-q23, and transcription is predominantly stimulated by interleukin (IL)-6 and IL-1β, which are produced in various cell types, including inflammatory cells (such as T cells and macrophages), endothelial cells, fibroblasts and esophageal cancer cells (3). In cancer patients, plasma CRP levels are typically high compared with healthy subjects (4).

Single nucleotide polymorphisms (SNPs) are the most common type of variation in the human genome and may influence disease susceptibility by affecting sequence coding and splicing (5). Previous studies have investigated the association between *CRP* 3407A>G and 29A>T polymorphisms, and cancer risk (6–10). However, association data determined from these independent studies is relatively powerless; therefore, the present study conducted a meta-analysis of all eligible published studies, and the effect of *CRP* 3407 A>G and 29 A>T polymorphisms on cancer risk was evaluated.

## Materials and methods

### Publication search

The PubMed database (http://www.ncbi.nlm.nih.gov/pubmed) was searched for relevant articles published prior to March 25, 2014. The following keywords were used: C-reactive protein or *CRP*, polymorphism and cancer. Additional studies were identified by conducting a manual search of the reference lists of reviews and the retrieved studies.

### Inclusion and exclusion criteria

All of the included studies met the following criteria: i) The association between *CRP* 3407 A>G and 29 A>T polymorphisms, and cancer risk were evaluated; ii) the studies were case-control; and iii) the number of individual genotypes for *CRP* 3407 A>G and 29 A>T polymorphisms in cancer cases and controls, respectively, were stated. In addition, the exclusion criteria included: i) Original or insufficient data regarding the individual genotypes for *CRP* 3407A>G and 29A>T polymorphisms in cancer cases and controls; ii) review articles and case studies focused on the prognosis associated with genetic polymorphism and iii) duplicated publications.

### Data extraction

Two independent researchers systematically extracted the relevant data from all of the studies that met the inclusion criteria using a standardized form. The following information was collected: First author’s name, publication year, country, ethnicity, cancer type and total number of genotyped cases and controls. If the study reported more than one ethnicity, the population was termed mixed.

### Statistical analysis

Crude odds ratios (ORs) and their corresponding 95% confidence intervals (CIs) were used to assess the strength of association between the *CRP* 3407 A>G and 29 A>T polymorphisms, and the risk of developing cancer. The pooled ORs were determined for recessive (GG vs. GA + AA; TT vs. TA + AA), dominant (GG + GA vs. AA; TA+TT vs. AA), co-dominant [(GG vs. AA; GA vs. AA), (TT vs. AA; TA vs. AA)] and additive (G vs. A; T vs. A) models. Furthermore, between-study heterogeneity was assessed by calculating the Q-statistic using the χ^2^ test and P_h_<0.05 was considered to indicate statistically significant heterogeneity. If the results were heterogeneous, the pooled ORs were calculated using the random-effect model; if the results were not heterogeneous, a fixed-effect model was used. In addition, a Begg’s funnel plot was constructed to examine any potential publication bias, and sensitivity analysis was performed to estimate the influence of excluding each individual study on the summary OR values. All statistical data were analyzed using Stata software (version 12.0; StataCorp LP, College Station, TX, USA).

## Results

### Study characteristics and quantitative synthesis

**Figure 1:**
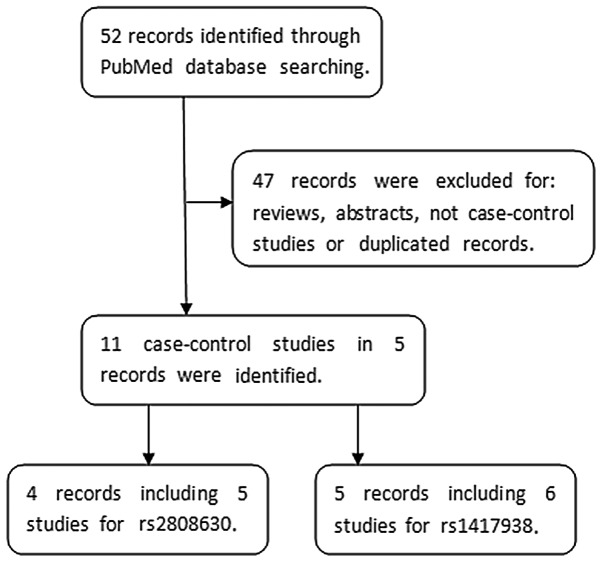
Flow chart of the study selection.

A flow chart of the study selection process is indicated in Fig. 1. A total of 52 studies regarding *CRP* polymorphisms with respect to cancer were identified; however, following a review of the titles, abstracts and articles, the inclusion and exclusion criteria identified five 3407 A>G polymorphism studies involving 888 cases and 3,167 controls, and six 29A>T polymorphism studies involving 3,110 cases and 5,951 controls for inclusion in the present meta-analysis (Table I).

**Figure 2:**
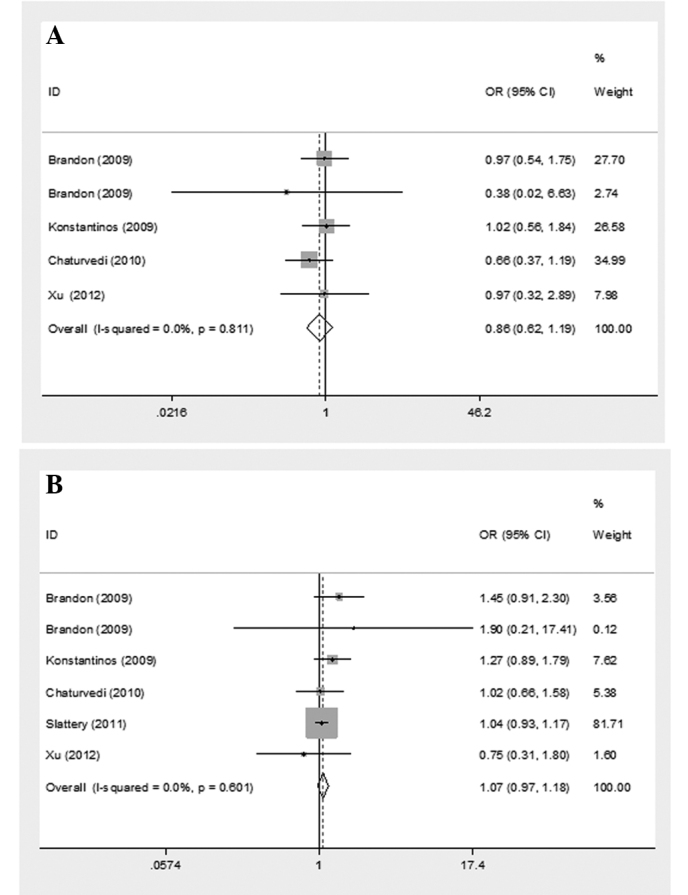
Forest plot describing the meta-analysis using the recessive model to determine the association between the *CRP* (A) 3407 A>G (GG vs. GA+AA) and (B) 29 A>T (TT vs. TA+AA) polymorphisms and the risk of developing cancer. ID, identification; OR, odds ratio; CI, confidence interval.

Evaluation of the association between *CRP* 3407 A>G and 29 A>T polymorphisms and cancer risk are indicated in Fig. 2 and Table II. Overall, when all of the eligible studies were pooled, no significant associations were observed between the *CRP* 3407 A>G polymorphism and overall cancer risk [GG vs. GA+AA, (OR, 0.86; 95% CI, 0.62–1.19; P_h_=0.81); GG+GA vs. AA, (OR, 1.09; 95% CI, 0.93–1.28; P_h_=0.61); GG vs. AA, (OR, 0.91; 95% CI, 0.65–1.27; P_h_=0.71); GA vs. AA, (OR, 1.13; 95% CI, 0.95–1.34; P_h_=0.66); and G vs. A, (OR, 1.03; 95% CI, 0.90–1.17; P_h_=0.63)] or the 29 A>T polymorphism and overall cancer risk [TT vs. TA+AA, (OR, 1.07; 95% CI, 0.97–1.18; P_h_=0.60; TA+TT vs. AA, OR, 1.03; 95% CI, 0.90–1.17; P_h_=0.74); TT vs. AA, (OR, 1.11; 95% CI, 0.94–1.31; P_h_=0.49); TA vs. AA, (OR. 1.00; 95% CI, 0.87–1.15; P_h_=0.86); and T vs. A, (OR, 1.04; 95% CI, 0.97–1.12; P_h_=0.64)]. In the subgroup analysis by cancer type, the results also indicated that *CRP* 3407 A>G and 29 A>T polymorphisms were not associated with prostate, lung and colorectal cancer.

### Sensitivity analysis and publication bias

**Figure 3:**
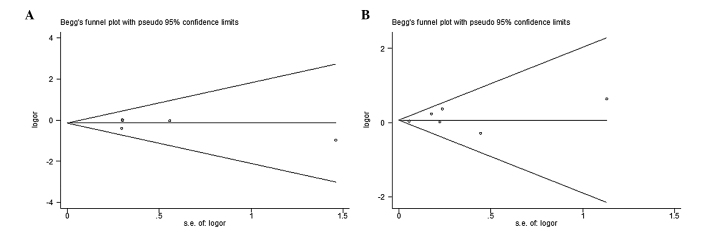
Funnel plot describing the meta-analysis of the association between the *CRP* (A) 3407A>G (GG vs. GA+AA) and (B) 29A>T (TT vs. TA+AA)polymorphisms and the risk of developing cancer.

The OR was not statistically different when excluding each individual study by sequence, demonstrating that no individual study significantly affected the pooled OR and clarifying the stability of the results of the present meta-analysis. The shapes of the Begg’s funnel plots, constructed to assess possible publication biases of the included studies, did not reveal any evidence of obvious asymmetry, which indicates a lack of publication bias (Fig. 3).

## Discussion

To the best of our knowledge, the present study is the first meta-analysis to comprehensively assess the association between *CRP* 3407 A>G and 29 A>T polymorphisms and cancer risk. In the present study, no association was identified between *CRP* 3407 A>G and 29 A>T polymorphisms and the overall cancer risk in all of the genetic models investigated. In the subgroup analysis based on cancer types, no association was identified between these polymorphisms, and prostate, lung and colorectal cancer. These findings indicate that *CRP* 3407 A>G and 29 A>T polymorphisms may not be risk factors for the development of cancer.

However, a number of limitations should be considered when interpreting the results of the present analysis. Firstly, the present results were based on unadjusted estimates, whereas a more precise analysis may have been conducted if individual data were available. Secondly, the relatively small subpopulation samples may have reduced the statistical power of the analysis; therefore, additional studies with a larger sample size are required to fully understand the association. Thirdly, the present analysis did not consider the possibility of gene-environment interactions, which may have influenced the effect of the polymorphisms on cancer risk.

In conclusion, the present meta-analysis indicates that *CRP* 3407 A>G and 29 A>T polymorphisms are not associated with cancer risk, particularly in prostate, lung and colorectal cancer.

## Figures and Tables

**Table I:** Characteristics of eligible studies investigated in the present meta-analysis.

A, rs2808630 (3407A>G) polymorphism										

					Case			Control		
						
Reference	Year	Country	Ethnicity	Cancer type	AA	AG	GG	AA	AG	GG
Pierce *et al* (6)	2009	USA	Caucasian	Prostate	89	73	13	1026	760	148
Pierce *et al* (6)	2009	USA	African	Prostate	24	16	0	184	107	9
Tsilidis *et al* (7)	2009	USA	Caucasian	Colorectal	96	84	19	204	124	34
Chaturvedi *et al* (8)	2010	USA	Mixed	Lung	206	153	19	242	172	33
Xu *et al* (9)	2012	China	Asian	Lung	52	38	6	65	51	8

B, rs1417938 (29A>T) polymorphism										

					Case			Control		
						
Reference	Year	Country	Ethnicity	Cancer type	AA	AT	TT	AA	AT	TT

Pierce *et al* (6)	2009	USA	Caucasian	Prostate	83	69	23	921	830	183
Pierce *et al* (6)	2009	USA	African	Prostate	31	8	1	230	66	4
Tsilidis *et al* (7)	2009	USA	Caucasian	Colorectal	15	74	109	43	140	177
Chaturvedi *et al* (8)	2010	USA	Mixed	Lung	186	149	42	221	177	49
Slattery (10)	2011	USA	Mixed	Colorectal	186	918	1120	238	1175	1373
Xu (9)	2012	China	Asian	Lung	49	38	9	59	50	15

**Table II:** Meta-analysis of the associations between the *CRP* gene polymorphisms and cancer risk in all of the possible genetic models.

A, rs2808630 (3407 A>G) polymorphism										

	GG vs. GA+AA		GG+GA vs. AA		GG vs. AA		GA vs. AA		G vs. A	
					
Cancer type	OR (95% CI)	P_h_-value	OR (95% CI)	P_h_-value	OR (95% CI)	P_h_-value	OR (95% CI)	P_h_-value	OR (95% CI)	P_h_-value
Prostate	0.92 (0.51–1.63)	0.53	1.09 (0.82–1.44)	0.93	0.96 (0.53–1.73)	0.53	1.11 (0.83–1.49)	0.93	1.03 (0.83–1.29)	0.76
Colorectal	0.72 (0.43–1.20)	0.55	0.97 (0.76–1.24)	0.86	0.73 (0.43–1.23)	0.61	1.02 (0.79–1.32)	0.72	0.94 (0.77–1.14)	0.93
Lung	1.02 (0.56–1.84)		1.39 (0.98–1.96)		1.19 (0.64–2.19)		1.44 (1.00–2.08)		1.22 (0.94–1.60)	
Total	0.86 (0.62–1.19)	0.81	1.09 (0.93–1.28)	0.61	0.91 (0.65–1.27)	0.71	1.13 (0.95–1.34)	0.66	1.03 (0.90–1.17)	0.63

B, rs1417938 (29 A>T) polymorphism										

	TT vs. TA+AA		TA+TT vs. AA		TT vs. AA		TA vs. AA		T vs. A	
					
Cancer type	OR (95% CI)	P_h_-value	OR (95% CI)	P_h_-value	OR (95% CI)	P_h_-value	OR (95% CI)	P_h_-value	OR (95% CI)	P_h_-value

Prostate	1.46 (0.93–2.30)	0.82	1.00 (0.75–1.33)	0.90	1.41 (0.88–2.27)	0.81	0.92 (0.68–1.25)	0.96	1.09 (0.87–1.35)	0.85
Colorectal	1.06 (0.96–1.18)	0.30	1.08 (0.89–1.30)	0.15	1.10 (0.91–1.34)	0.12	1.04 (0.85–1.27)	0.23	1.05 (0.97–1.14)	0.13
Lung	0.96 (0.65–1.41)	0.54	0.97 (0.76–1.24)	0.64	0.95 (0.63–1.43)	0.51	0.98 (0.76–1.27)	0.79	0.98 (0.81–1.18)	0.52
Total	1.07 (0.97–1.18)	0.60	1.03 (0.90–1.17)	0.74	1.11 (0.94–1.31)	0.49	1.00 (0.87–1.15)	0.86	1.04 (0.97–1.12)	0.64

P_h_ represents the difference in heterogeneity within each polymorphism. OR, odds ratio; CI, confidence interval.
